# Targeted metabolomic approach in men with carotid plaque

**DOI:** 10.1371/journal.pone.0200547

**Published:** 2018-07-16

**Authors:** Teresa Auguet, Gemma Aragonès, Marina Colom, Carmen Aguilar, Vicente Martín-Paredero, Núria Canela, Xavier Ruyra, Cristóbal Richart

**Affiliations:** 1 Grup de Recerca GEMMAIR (AGAUR)- Medicina Aplicada, Departament de Medicina i Cirurgia, Universitat Rovira i Virgili (URV), Institut d’Investigació Sanitària Pere Virgili (IISPV), Tarragona, Spain; 2 Servei de Medicina Interna, Hospital Universitari Joan XXIII, Tarragona, Spain; 3 Servei Angiologia i Cirurgia Vascular, Hospital Universitari Joan XXIII, Tarragona, Spain; 4 Unitat Mixta Centre for Omic Sciences, Universitat Rovira i Virgili–Eurecat, Reus, Spain; 5 Servei de Cirurgia Cardíaca, Hospital Germans Trias i Pujol, Badalona, Spain; Max Delbrueck Center for Molecular Medicine, GERMANY

## Abstract

**Background:**

The aim of the study was to analyse the presence of several metabolites related to atherosclerosis in the plasma of patients with unstable carotid plaque and in the plasma of healthy subjects.

**Materials and methods:**

We included 20 patients who had undergone carotid endarterectomy and 20 healthy subjects as a control group. All the subjects recruited were male. We used a metabolomic approach with liquid chromatography coupled to mass spectrometry to evaluate plasma metabolite levels in the metabolic pathway involved in the progression of atherosclerotic plaque.

**Results:**

We observed that circulating levels of 20-HETE were significantly higher in patients with atheroma plaque than in healthy subjects (*p* = 0.018). No differences were found with regard to the other metabolites analysed. We also conducted a random forest analysis and found that 20-HETE was the main differentiator in the list of selected metabolites. In addition, plasma levels of 20-HETE correlated positively with body mass index (r = 0.427, *p* = 0.007) and diastolic blood pressure (r = 0.365, *p* = 0.028).

**Conclusion:**

This study confirms that of all the molecules studied only 20-HETE is related to carotid plaque. Further studies are needed to compare patients with stable carotid plaque vs. patients with unstable carotid plaque in order to confirm that 20-HETE could be a potential factor related to carotid plaque.

## Introduction

Atherosclerosis is a systemic disease and one of the leading causes of death in the Western world [1]. Rupture of the atherosclerotic plaque can generate a thrombus that partially or completely occludes normal blood flow. Patients with cerebrovascular symptoms caused by carotid atherosclerosis are at high risk of an imminent life-threatening stroke. Moreover, a high percentage of stroke patients have been previously asymptomatic [2]. Therefore, there is a clinical need to identify biological markers that can define the risk of carotid plaque rupture.

Metabolomics is often used to study small endogenous molecules or metabolites in biological samples. Several risk factors for atherosclerosis induce the activation of pathways which generate a complex interplay between metabolites absorbed from or released in blood, which will consequently modify vascular homeostasis. Nowadays, metabolic profiling in cardiovascular disease has been predominately used in human studies and animal models [3,4]. However, metabolic profiling technologies have only been used in a limited number of cardiovascular disease studies. This could be an innovative way to investigate the molecular basis of vascular disease. In this regard, recent metabolic phenotyping and profiling studies have obtained promising results on the atherosclerotic plaque of both carotid and coronary arteries and peripheral artery diseases [5–7].

Platelet activation results in the release of biologically active microparticles such as thromboxane A2, ADP, thrombin or platelet activating factor, released from endothelial cells, polymorphonuclear leukocytes or monocytes. It has been shown that dysregulation of platelet activity is linked to the progression of atherosclerosis and mainly involves platelet aggregation and a blood flow decrement in the vascular endothelium. The major platelet activation pathways mediated by agonists involve the arachidonic acid, adenosine diphosphate and nitric oxide pathways, and action of free radicals on molecules related to platelet aggregation. Regarding adenosine diphosphate pathway, ADP and ATP regulate platelet activation resulting in shape change, aggregation, thromboxane A2 production, and release of granule contents. ADP also causes a number of intracellular events including inhibition of adenylyl cyclase, mobilization of calcium from intracellular stores, and rapid calcium influx in platelets [8,9]. On the other hand, eicosanoids can stimulate or inhibit platelet reactivity [10]. Platelet cyclooxygenase (COX)-1 generated thromboxane (TX) A2 is the primary prostanoid that stimulates platelet aggregation; its action is counter-balanced by prostaglandin (PG)I2 (prostacyclin), a product of vascular COX. PGD2, PGE2, and HETEs, are other prostanoid modulators of platelet activity. In fact, some studies had studied HETE levels, which are oxidized esters of arachidonic acid, and had described them to be associate with plaque instability [11].

On the basis of this and our previous studies on adipocytokine levels in atherosclerotic secretomes and the proteomic profile of unstable carotid plaque [12,13], the aim of this study was to analyse the presence of several metabolites in metabolic pathways involved in atherosclerotic disease. To achieve our objective, we conducted a metabolomic study on plasma samples from patients with carotid atheroma plaque and healthy subjects. Ultra-performance liquid chromatography-triple-quadrupole-mass spectrometry (QqQ/MS) was used to make a full assessment of the metabolites.

## Material and methods

### Subjects/Samples

The study was approved by the institutional review board “Comitè d’Ètica d’Investigació Clínica, Hospital Universitari de Sant Joan de Reus” (10-04-29/4proj3). All participants gave written informed consent to participate in medical research. All the subjects recruited were male. We conducted the study only in men because the majority of the patients in the Universitary Hospital who undergo carotid endarterectomy were men and in order to avoid interference of several confounding factors such as gender. We selected 20 patients (mean age 66.88 ± 4.99years) who underwent Carotid Endarterectomy (CE) at the Angiology and Vascular Surgery Unit of the Hospital Universitari Joan XXIII (Tarragona, Spain). These patients had cerebrovascular ischemia and internal carotid artery stenosis >75%, diagnosed by colour Doppler assisted duplex imaging and arteriography. Patients were excluded if they had clinical coronary and peripheral arterial disease, diabetes mellitus, severe obesity, malignancies, renal failure or poorly controlled hypertension (SBP>140, DBP>90). Patients who were concurrently being treated with fibrates, angiotensin-renin inhibitors and antiplatelet therapy were also excluded.

A total of 20 clinically healthy men (mean age 69.43 ± 3.21years) were also recruited for the control group. The exclusion criteria were as follows: cardiovascular event, cardiac arrhythmias or valve pathologies, cerebral or peripheral atherosclerotic disease, diabetes mellitus, malignancies, renal failure or inflammatory conditions. The control subjects did not regularly use any medications.

### Clinical and biochemical assessments

A complete anthropometric, physical examination and biochemical analysis was carried out on each patient. Body height and weight were measured with the patient standing in light clothes and without shoes. Body mass index (BMI) was calculated as body weight divided by height squared (kg/m^2^).

Blood samples were obtained from each individual after overnight fasting. Plasma was obtained by standard protocols and preserved at -80°C until use. Laboratory studies included glucose, insulin, glycated haemoglobin (HbA1c), total cholesterol, high-density lipoprotein cholesterol (HDL-C), low-density lipoprotein cholesterol (LDL-C) and triglycerides, all of which were analysed using a conventional automated analyser. Insulin resistance (IR) was estimated using the homeostatic model assessment of IR (HOMA2-IR).

### Sample preparation and analysis

#### Analysis of adenosine triphosphate (ATP), adenosine diphosphate (ADP), 3’,5’-cyclic-adenosine monophosphate (3’,5’-cyclic-AMP), and 3’,5’-cyclic-guanosine monophosphate (3’,5’-cyclic-GMP)

To analyze ATP, ADP, 3’,5’-cyclic-AMP and 3’,5’-cyclic-GMP we used the method described by Zhang et al. [14]. Five microliters of internal standard (^13^C_10_^15^N_5_ -ATP) was added to a plasma sample (100 μL) and thoroughly mixed. Then, a sample was extracted with 300 μL acetonitrile, shaken for 10 min and centrifuged at 15000 rpm at 4°C for 15 min. Three hundred microliters of supernatant was evaporated under nitrogen to dryness. The residue was reconstituted with 75 μL of H_2_O and injected.

The analyses were conducted on an Agilent 1290 UHPLC chromatograph coupled to a triple quadrupole (QqQ) 6495 Series with an ESI interface (Agilent Technologies, Waldbronn, Germany). The chromatographic column used was Acquity UPLC HSS T3 (2.1 mm x 100mm, 1.8 μm, Waters, Milford MA, USA). The chromatographic separation was performed in gradient elution with ultrapure water, and 10 mM ammonium acetate (A) and acetonitrile (B) as the mobile phases, at a flow rate of 0.5 mL/min. The gradient was as follows: 0–1.0 min, 0% B, isocratic; 1.0–5.0 min, 50% B, linear gradient; 5.0–6.0 min, 100% B linear gradient, 6.0–9.0 min, 100% B, isocratic and 9.0–10.0, 0% B, linear gradient and 10.0–12.0, 0% B, isocratic. Mass spectrometric detection was performed by multiple reaction monitoring (MRM) in positive ESI mode and several transitions for each compound were acquired. The transitions monitored were: 508.0>135.9 and 508.0>410.2 for ATP (rt: 0.7 min), 428.0>135.9 and 428.0>348.1 for ADP (rt: 0.9 min), 330.1.>136.0, 330.1>119.0 and 330.1>96.9 for cAMP (rt: 2.7 min), 346.1>152.1, 346.1>135.1 and 346.1>110.0 for cGMP (rt: 2.5 min) and 523.1>146.0 and 523.1>425.1 for ^13^C_10_^15^N5 –ATP (rt: 0.7 min). The ESI parameters were as follows: gas temperature (240°C), gas flow rate (13 L/min), nebuliser pressure (10 psi), sheath gas temperature (400°C), sheath gas flow (10 L/min), capillary voltage (3500V) and nozzle voltage (2000V).

#### Analysis of arachidonic acid (AA), 12-hydroxyeicosatetranoic acid (12-HETE), 15- hydroxyeicosatetranoic acid (15-HETE), 20- hydroxyeicosatetranoic acid (20 HETE), 11(12)-dihydroxyeicosatetranoic acid (11(12)-DiHETE) and 14(15)-dihydroxyeicosatetranoic acid (14(15)-DiHETE)

Two sample preparation methodologies were used and validated for the analysis of AA, 12-HETE, 15-HETE, 20-HETE, 11(12)-DiHETE and 14(15)-DiHETE in plasma [15]. First, to extract AA and 12 HETE a protein precipitation was applied. To 25 μL of plasma, 100 μL of methanol and 5 μL of internal standard (AA-*d8*) were added. Then, the mix was shaken for 30 minutes and incubated at -20 °C for 30 minutes. Afterwards, the Eppendorf was centrifuged at 15000 rpm, at 4°C for 15 minutes, and then 100 μL of the supernatant was collected into an injection vial. To analyze 15-HETE, 20 HETE, 11(12)-DiHET and 14(15)-DiHET a modified version of the method described by Mangal et al. [1] was used. Five microliters of internal standard was added to plasma samples (250 μL) and thoroughly mixed. The eicosanoids were extracted with 1.25 mL chloroform/isopropanol (2:1); and a second extraction step was performed by adding MTBE (1.25 mL) to the aqueous layer, resulting from the previous extraction. Both organic layers were mixed and evaporated to dryness at room temperature under nitrogen. The dried extract was then reconstituted in 50 μL of methanol.

The analyses were conducted on an Agilent 1290 UHPLC chromatograph coupled to a triple quadrupole (QqQ) 6495 Series instrument, with an ESI interface (Agilent Technologies, Waldbronn, Germany). The chromatographic column used was Kinetex EVO C18 (2.1 mm x 150mm, 2.6 μm, Phenomenex (Torrance, CA, USA)). The chromatographic separation was performed in gradient elution with ultrapure water with formic acid (0,025%) and with acetonitrile (B) as the mobile phases, at a flow rate of 0.4 mL/min. The gradient was as follows: 0–0.1 min, 35% B, isocratic; 0.1–2.0 min, 47% B, linear gradient; 2.0–3.5 min, 54% B, linear gradient; 3.5–4.5 min, 55% B, linear gradient; 4.5–9 min, 60% B, linear gradient; 9–13.5 min, 70% B, linear gradient; 13.5–14.5 min, 80% B, linear gradient; 14.5–15.5 min, 100% B, linear gradient; 15.5–17.5 min, 100% B, isocratic;17.5–18.0, 35% B, linear gradient and finally, 18.0–20.0, 35% B, isocratic. Mass spectrometric detection was performed by multiple reactions monitoring (MRM) in negative ESI mode and three transitions for each compound were acquired at their specific retention time. The transitions monitored were: 337.2>207.1 and 337.2>319.3 for 14(15)-DiHET (rt: 5.4 min), 337.2>167.0 and 337.2>319.3 for 11(12)-DiHET (rt: 5.7 min), 319.2>275.3, 319.2>301.3 and 319.2>289.2 for 20-HETE (rt: 6.6 min), 319.2>219.2, 319.2>301.3 and 319.2>175.0 for 15-HETE (rt: 7.6 min), 319.2>179.2, 319.2>301.3 and 319.2>257.2 for 12-HETE (rt: 8.2 min), 303.2>259.2, 303.2>205.1 and 303.2>58.7 for AA (rt: 15.1 min) and 311.3>267.3 for AA-*d8* (rt: 14.9 min). The ESI parameters were as follows: gas temperature (150°C), gas flow rate (17 L/min), nebuliser pressure (35 psi), sheath gas temperature (300°C), sheath gas flow (12 L/min), capillary voltage (2500V) and nozzle voltage (1000V).

### Statistical analysis

All the values reported are expressed as mean ± standard deviation (SD) and were analysed using the Windows SPSS/PC+ statistical package (version 22.0; SPSS, Chicago, IL, USA) and the program ‘R’ (http://cran.r-project.org). Differences between groups were calculated using Student’s t test. The strength of association between variables was calculated using the Spearman Rho correlation test for non-parametric contrasts. In addition, the fold change of each variable was represented with Heat Map (univariate test). Random Forests is a supervised classification technique based on an ensemble of decision trees. This method was used as a multivariate test that provides an unbiased estimate of how well one can predict sample classes in a new data set (prediction accuracy) and a selection of the variables that make the largest contributions to the classification. *P* values < 0.05 were considered to be statistically significant.

## Results

### Baseline characteristics of subjects

The clinical characteristics and biochemical parameters of the population studied are shown in **Table 1**. The two groups were comparable in terms of age (*p* = 0.292) and BMI (*p* = 0.266). All subjects were male. Biochemical analyses indicated that patients who underwent CE had significantly higher levels of fasting glucose and HbA1c (*p* = 0.018 and *p* = 0.003; respectively) and lower levels of HDL-C (*p* = 0.003) than healthy subjects. However, levels of total cholesterol and LDL-C were significantly decreased in CE patients (*p*<0.001) since 75% of them received lipid-lowering therapy. Our patients were in lipid lowering treatment only with statins. Patients who received fibrates and other hypolipemiant drugs were excluded from the study.

**Table 1:** Clinical baseline characteristics of the population studied.

	Serum control group (n = 20) Mean ± SD	Patients who underwent carotid endarterectomy (n = 20) Mean ± SD	p-value
**Age (years)**	69.43 ± 3.21	66.88 ± 4.99	0.292
**Weight (kg)**	74.15 ± 8.64	74.71 ± 12.78	0.872
**BMI (kg/m**^**2**^**)**	25.95 ± 3.13	27.18 ± 3.68	0.266
**Glucose (mg/dl)**	87.31 ± 18.31	106.58 ± 26.08	**0.018**
**HbA1c (%)**	5.26 ± 0.38	6.11 ± 1.00	**0.003**
**Insulin (mUI/L)**	6.94 ± 4.15	10.76 ± 10.09	0.143
**HOMA2-IR**	0.93 ± 0.53	1.42 ± 1.26	0.139
**Triglycerides (mg/dL)**	92.53 ± 38.19	123.74 ± 72.45	0.112
**Cholesterol (mg/dl)**	176.84 ± 42.37	115.89 ± 32.19	**<0.001**
**HDL-C (mg/dL)**	46.80 ± 20.41	29.47 ± 5.42	**0.003**
**LDL-C (mg/dL)**	111.52 ± 29.36	64.23 ± 24.02	**<0.001**
**DBP (mmHg)**	76.94 ± 9.26	73.72 ± 14.82	0.439
**SBP (mmHg)**	128.94 ± 8.79	130.39 ± 10.22	0.498
**Lipid-lowering therapy (%)**	-	75	-
**Antihypertensive therapy (%)**	-	50	-

BMI. body mass index; HbA1c. glycosylated haemoglobin; HDL-C. high density lipoprotein; HOMA2-IR. homeostatic model assessment 2-insulin resistance; LDL-C. low density lipoprotein; DBP. diastolic blood pressure; SBP. systolic blood pressure. Data are expressed as mean ± SD. *P*<0.05 are considered statistically significant. HOMA-2 is calculated using the HOMA Calculator version 2.2.2 (http://www.dtu.ox.ac.uk).

### Plasma analysis of selected metabolites

In order to assess potential biomarkers for atheromatous plaque disease, we focused on previously described metabolites that are associated with metabolic pathways involved in atherosclerotic disease [13]. In one of our previous studies, we conducted pathway analysis on proteomic data using the ConsensusPathDB-human Platform. Interestingly, we found metabolic pathways involved in atherosclerotic progression such as platelet activation. In this context, in the present study we decided to go a step further and explore potential biomarkers of atherosclerosis. Using a targeted metabolomic approach, we determined the plasma levels of ATP, ADP, 3’-5’-cyclic-AMP, 3’-5’-cyclic-GMP, AA, 12-HETE, 15-HETE, 20-HETE, 11(12)-DiHETE and 14(15)-DiHETE in patients who underwent CE and healthy subjects.

**Fig 1:**
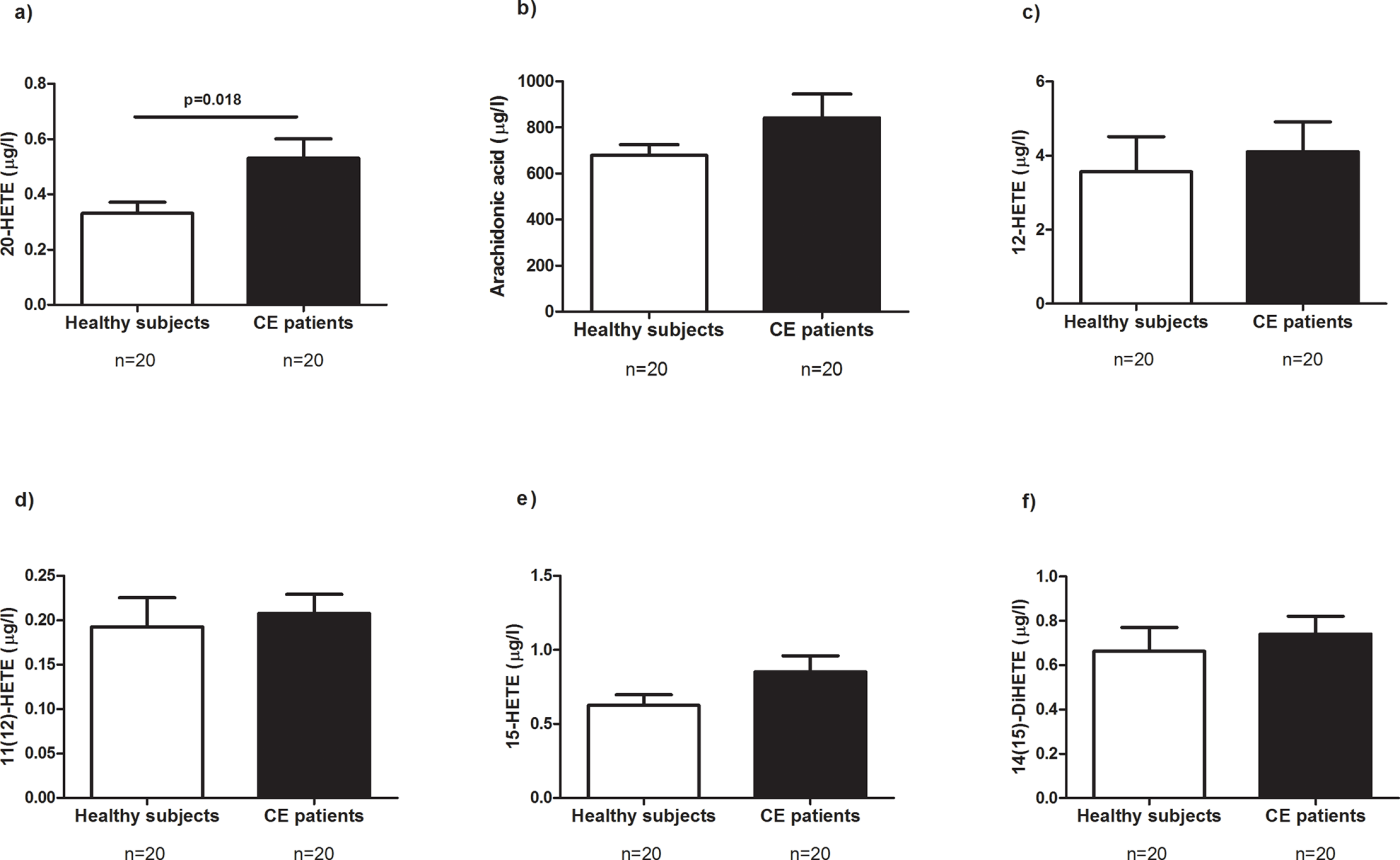
**Plasma levels of metabolites in healthy subjects and in patients who underwent carotid endarterectomy (CE):** (**a**) 20-HETE; (**b**) Arachidonic acid; (**c**) 12-HETE; (**d**) 11(12)-DiHETE; (**e**) 15-HETE; (**f**) 14(15)-DiHETE. *p*<0.05 was considered to be statistically significant. HETE, hydroxyeicosatetranoic acid.

**Fig 2:**
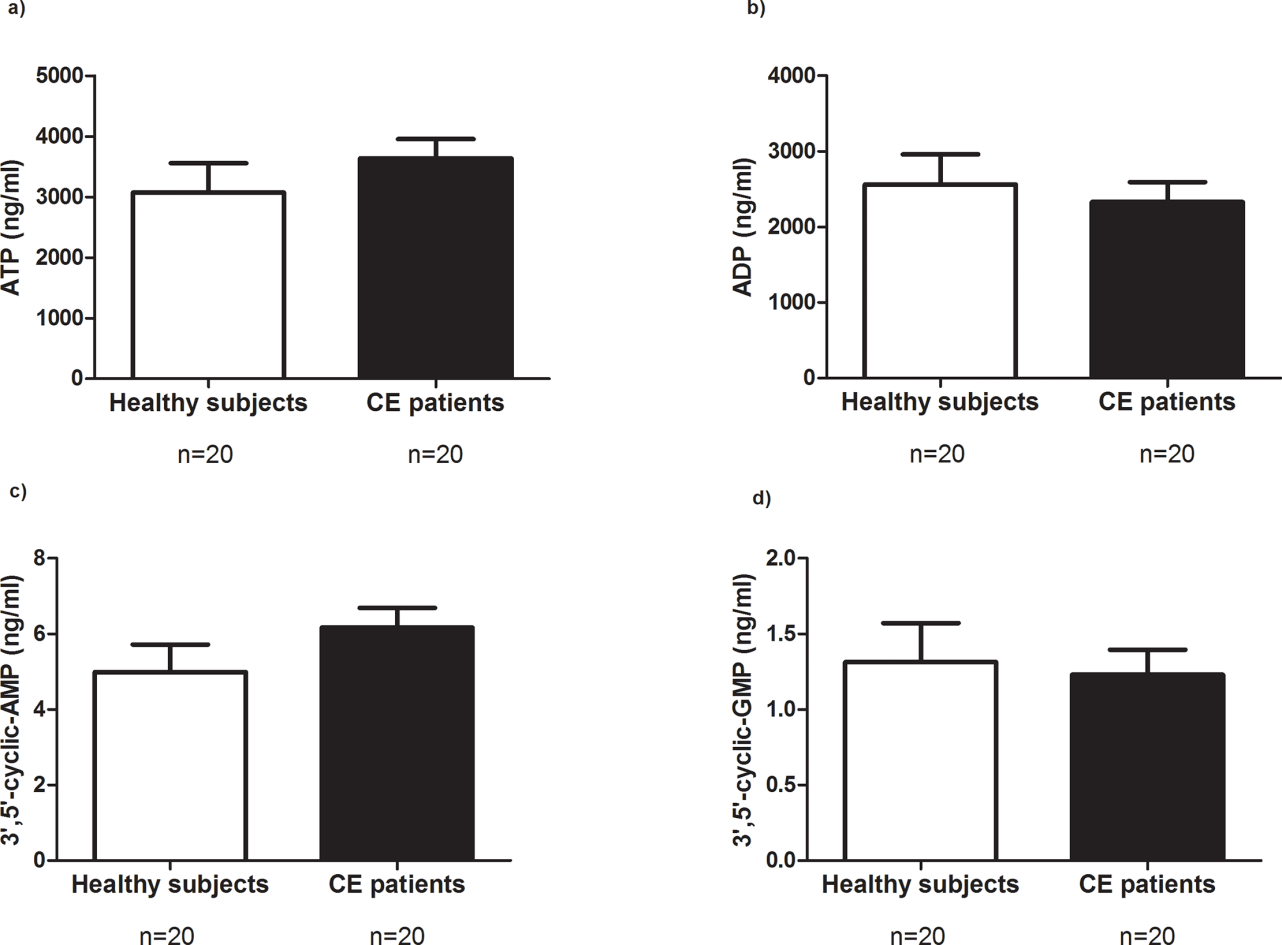
**Plasma levels of metabolites in healthy subjects and in patients who underwent carotid endarterectomy (CE):** (**a**) ATP; (**b**) ADP; (**c**) 3’,5’-cyclic-AMP; (**d**) 3’,5’-cyclic-GMP. *p*<0.05 was considered to be statistically significant. ATP, adenosine triphosphate; ADP, adenosine diphosphate; AMP, adenosine monophosphate, GMP, guanosine monophosphate.

First, we used univariate statistics to define specific potential biomarkers. Of all molecules analysed, 20-HETE was the only metabolite that was differently expressed in plasma samples. Specifically, 20-HETE levels were significantly higher in the group of CE patients than in healthy subjects (**Fig 1A**, *p* = 0.018). No differences were found with regard to the other metabolites analysed (**Fig 1B–1F** and **Fig 2A–2D**).

**Fig 3:**
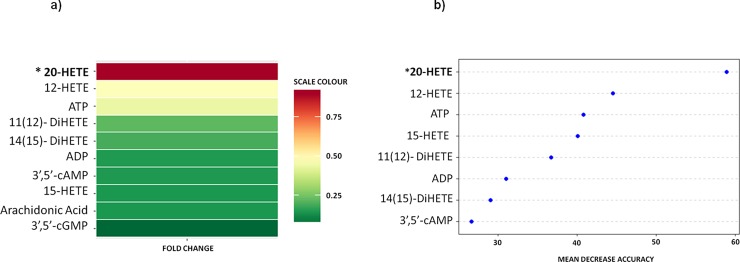
(**a**) The heat map represents the fold-change for each metabolite relative to control subjects (red, greater; green, lower). (**b**) Random (decision) Forest analysis was used as implemented in the R package software as a framework to create a large number of particular models built around the presence or absence of atheroma plaque.

Then, we created a heat map that represents the fold-change for each selected metabolite relative to control subjects (**Fig 3A**). Of all the metabolites, we found that 20-HETE had the highest fold-change. It is important to highlight that 20-HETE was the most significant in the univariate test. Moreover, to study changes in the plasma concentration of metabolites, a random forest test was applied. The analysis distinguished between patients with or without carotid atheroma plaque with an accuracy of 60% and revealed plasma 20-HETE as the primary differentiator in a list of metabolites ranked in order of importance in the classification scheme (**Fig 3B**).

Regarding correlation analysis, 20-HETE circulating levels correlated positively with BMI and diastolic blood pressure (r = 0.427, *p* = 0.007; r = 0.365, *p* = 0.028; respectively, **Table 2**) in the population studied.

**Table 2:** Correlations between the levels of 20-HETE and clinical parameters.

Variables	20-HETE		
	N	r	p-value
BMI	40	**0.427**	**0.007**
Glucose	40	0.045	0.796
HbA1c	39	-0.019	0.916
Insulin	40	0.051	0.767
Cholesterol	39	-0.258	0.114
HDL-C	39	-0.256	0.131
LDL-C	39	-0.302	0.078
Triglycerides	39	-0.267	0.115
SBP	36	0.241	0.157
DBP	36	**0.365**	**0.028**

BMI, body mass index; HbA1c, glycosylated haemoglobin; HDL-C, high density lipoprotein; LDL-C, low density lipoprotein; SBP, systolic blood pressure; DBP, diastolic blood pressure. The strength of association between variables was calculated using Spearman’s *r* correlation test. *P*<0.05 is considered statistically significant.

## Discussion

In a previous proteomic study, we provided a subset of identified proteins that were differently expressed in carotid atheroma secretomes and we determined the metabolic pathways involved in atherosclerotic progression. In this context, in the present research we decided to go a step further and explore potential metabolites related to atherosclerosis. So we decided to study selected metabolites from platelet activation pathways. Some of the main pathways involve in platelet activation/aggregation are the adenosine diphosphate and arachidonic acid metabolism. Taken this into account, we decided to study metabolites of these pathways. We did not include other metabolites related that could be modified by pharmacological treatments and clinical and immunological factors. Of all the molecules studied related to platelet activation pathway, we found that only 20-HETE had increased plasma levels in patients who underwent carotid endarterectomy. Our findings confirmed a clear relationship between carotid atherosclerosis plaque and plasma levels of 20-HETE. Due to the fact that our study was cross-sectional, it allowed us to detect correlations but not to formulate predictions. To assess the clinical relevance of plasma 20-HETE levels, prospective studies are needed.

20-Hydroxyeicosatetraenoic acid (20-HETE), an arachidonic metabolite, is a potent vasoactive cytochrome (CYP) eicosanoid and a key constituent of microcirculation. It is present in such organs and tissues as liver, kidney, heart, lung and brain. Studies have shown that CYP epoxygenases influence vascular inflammation and atherosclerosis, which make an important contribution to the progression of the disease [16,17]. Moreover, epoxyeicosatrienoic acids (EETs) have been shown to attenuate vascular inflammation and decrease endothelial cell adhesion molecule expression [18]. In agreement with us, some authors have already shown in humans that 20-HETE was associated with hypertension and BMI [19–22]. In addition, Ward et al. suggested that 20-HETE plays a role in the pathophysiology of acute ischaemic stroke and subsequent clinical outcome [23]. According with us, recent study also showed that 20-HETE was associated with endothelial cell proinflammatory actions that promote atherosclerosis and vascular remodeling [24]. Among the proinflammatory endothelial cell changes that occur in response to 20-HETE are increased adhesion molecule expression and cytokine release [25]. Alterations in levels of CYP enzymes, 20-HETE or EETs contribute to endothelial dysfunction and cardiovascular diseases such as ischemic injury, hypertension and atherosclerosis [16,17].

Previous metabolic analysis of patients with carotid plaque and stroke revealed alterations in the CYP-eicosanoid profile. Plasma samples from an initial cohort of 131 transient ischemic attack (TIA) patients suggested metabolomic patterns that were specific to stroke recurrence subjects, non-stroke recurrence subjects and late stroke recurrence subjects [26]. In another study related to stroke, Vorkas PA et al [6] demonstrated enhanced downregulation of the β-oxidation pathway in symptomatic plaques. The metabolic signature of carotid plaque tissue from patients with cerebrovascular symptoms differed significantly from carotid plaque tissue from asymptomatic patients. The metabolic signatures identified showed they had the potential to become differential diagnostic biomarkers for symptomatic plaques. The limitation of this study was the number of patients studied, there were only five subjects per group. The use of metabolomics in stroke recurrence biomarker research could improve the predictive power of conventional predictors and may provide targets for pharmacotherapeutic intervention. Xing-Yang Y et al. [27] studied eleven single nucleotide polymorphisms (SNOs) of CYP genes and their plasma metabolites. Specifically, they analyzed 20-HETE, total epoxyeicosatrienoic acids (EET) and dihydroxyeicotatriennoic acids (DiHETEs) in 396 patients with ischemic stroke and carotid plaque. Some polymorphisms were associated with CYP plasma metabolite levels. Patients with echolucent plaque showed significantly higher levels of 20-HETE and DiHETEs but lower levels of EETs.

Our findings described that among all metabolites of the platelet activation/aggregation pathway analysed in a group of men with unstable carotid plaque vs a healthy control group, the only metabolite which showed the highest and most significant levels is 20-HETE. It is important to note that results could be considered with significant statistical power because we analysed our data by three statistically methods (univariate test, fold change and random forest test).

The major limitation of our study is the lack of a comparable group of patients with stable carotid plaque. Therefore, we could not conclude that 20-HETE was a potential factor related to plaque instability or atherosclerosis progression. Thus, further studies with large number of subjects subclassified by gender are needed to compare patients with stable carotid plaque vs patients with unstable carotid plaque.

In our point of view, our work is a preliminary study which suggests new metabolic clues about the importance of identifying interactions between metabolites and others physiopathological factors. The arterial carotid plaque is an important clinical problem with few metabolomic studies published. Hence, it seems interesting to publish our results on 20-HETE as a possible metabolite related to this pathology.
